# Modulation of viscoelastic fluid response to external body force

**DOI:** 10.1038/s41598-019-45612-2

**Published:** 2019-06-28

**Authors:** Meng Zhang, Wu Zhang, Zhengwei Wu, Yinan Shen, Huayin Wu, Jianping Cheng, Hongna Zhang, Fengchen Li, Weihua Cai

**Affiliations:** 1 School of Energy Science and Engineering, Harbin Institute of Technology, Harbin, 150001 China; 2 School of Engineering and Applied Science, Harvard University, Cambridge, 02138 United States; 3 Department of Biomedical Engineering and Biotechnology, University of Massachusetts Lowell, Lowell, MA 01854 United States

**Keywords:** Fluid dynamics, Applied physics

## Abstract

Transient flow responses of viscoelastic fluids to different external body forces are studied. As a non-Newtonian fluid, the viscoelastic fluid exhibits significant elastic response which does not raise in Newtonian fluid. Here, we investigate the transient response of a viscoelastic Poiseuille flow in a two-dimensional channel driven by external body forces in different forms. The velocity response is derived using the Oldroyd-B constitutive model in OpenFOAM. Responses in various forms like damped harmonic oscillation and periodic oscillation are induced and modulated depending on the fluid intrinsic properties like the viscosity and the elasticity. The external body forces like constant force, step force and square wave force are applied at the inlet of the channel. Through both time domain and frequency domain analysis on the fluid velocity response, it is revealed that the oscillation damping originates from the fluid viscosity while the oscillation frequency is dependent on the fluid elasticity. The velocity response of the applied square waves with different periods shows more flexible modulation signal types than constant force and step force. An innovative way is also developed to characterize the relaxation time of the viscoelastic fluid by modulating the frequency of the square wave force.

## Introduction

Fluid has been widely used as the medium for energy and force transmission in control systems named as fluidic devices. Such devices has been developed into functional dynamic elements like amplifiers and triodes, and exhibit logical operations similar as electronic components^1–5^. Much attention has been paid to the fluid logic component due to its advantages of no extra mechanical structure, low cost and high reliability. Its resistance to extreme conditions of shock, vibration or radiation is also desired for many applications.

Traditionally, fluidic logical functions are implemented in macro-scale devices at high Reynolds (*Re*) number flows by taking the advantage of the nonlinear property of Newtonian fluid^6^. While in microfluidic component, the logical function usually relies on the formation of droplets^7^, bubbles^8^ or circuits with valve switches^9^. For real application, these methods usually require complex control systems. On the other hand, realizing nonlinear behavior of Newtonian fluid for microfluidic components remains large challenging as extremely high pressure is required to achieve high *Re* number condition^10^. However, other studies^11–14^ show that viscoelastic fluid can induce nonlinear characteristic from its elastic property even at extremely low *Re* conditions^15–17^. Therefore, by using a viscoelastic fluid, a nonlinear microfluidic circuit can be obtained.

Based on the viscoelastic fluid, many microfluidic logical components are developed for microfluidics applications^18–20^. Microfluidic diodes, rectifiers and amplifier are three most common microfluidic circuit components functioning with viscoelastic fluid^20–22^. Grosiman *et al*. designed a bistable flip-flop memory and a flux stabilizer with viscoelastic fluid in a continuously curved sudden contraction structure^21^. Also, they proposed a microfluidic rectifier device^22^ using a nozzle and diffuser structure by taking the viscoelastic flow direction as the logic operation signal. It had been successfully used to characterize the viscoelastic rectifier^23–25^. The above studies mainly took the viscoelastic flow direction as the logic operation signal without investigating the response of fluid itself. Actually, the transient response of the viscoelastic fluid to external excitations will provide a new way to implement reliable microfluidic circuit components with dynamic modulation functions. The response of viscoelastic fluid has been previously studied for a start-up flow under an impulse or constant pressure gradient at rest^26–29^. The start-up flow for non-Newtonian fluid are particularly for the verification of the numerical methods, which are used for calculating transient flow responses^30–32^.

In this paper we propose to consider the viscoelastic fluid itself as a transducer medium so as to realize different microfluidic signals. Particularly, we investigate the velocity response in the two-dimensional fully-developed Poiseuille flow based on numerical simultions, which are realized by the open sourse OpenFOAM^33^. Both time domain and frequency domain of the velocity response are investigated for different excitation forces including the constant force, the step function force and the square wave force. The effect of varying fluid parameters like elasticity and viscosity on the response is also detailedly analyzed.

## Formulation and Numerical Method

### Physical model

**Figure 1:**
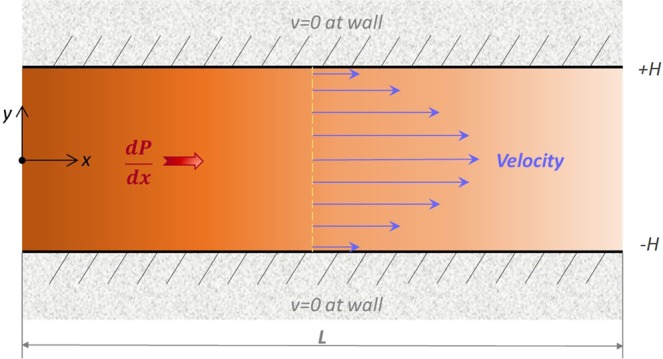
Schematic of two-dimensional Poiseuille flow.

This paper studies the velocity response of viscoelastic fluid to an applied external body force in a two-dimensional fully-developed Poiseuille flow model as shown in Fig. 1. The widths of both the inlet and the outlet 2*H* are set at a dimensionless number of 2; the flow distance *L* is set at 8. The two walls are in the non-slip boundary condition. An external body force is exerted in the *x*-direction.

### Governing equations for viscoelastic fluid flow

Firstly, we need to choose suitable constitutive models to represent the flow properties of viscoelastic fluids. There are various constitutive models being developed, among which two models are often used^34^: (1) limited linear models including Maxwell model, Jeffreys model and Generalized Maxwell model^35^, etc.; (2) nonlinear differential models including Convected Maxwell model, Convected Jeffreys model, Oldroyd 8-Constant model, White-Metzner Model, FENE (FENE-P) model and Giesekus model^36^, etc. In this study, we adopt the Oldroyd-B model, which is one of the Oldroyd 8-Constant models due to its simplicity. Based on the Oldroyd-B model, we build a new solver for viscoelastic fluid flow in OpenFOAM.

The dimensionless governing equations for incompressible viscoelastic fluid flow are expressed as::

1 $$\nabla \cdot {\bf{u}}=0$$

2 $$\frac{\partial {\bf{u}}}{\partial {\rm{t}}}+{\bf{u}}\cdot \nabla {\bf{u}}=-\,\nabla p+\nabla \cdot ({{\bf{T}}}^{s}+{{\bf{T}}}^{p})+{F}_{0}{\bf{i}}$$

where **u** is the velocity vector normalized by the maximum velocity U_max_; *p* is the pressure in the fluid and normalized by ρU_max_^2^, where ρ is density of the fluid; **T**^*s*^ is the viscous stress, and **T**^*p*^ is the elastic stress. **T**^*s*^ is described as ${{\bf{T}}}^{s}=\frac{\beta }{Re}[\nabla {\bf{u}}+{(\nabla {\bf{u}})}^{{\rm{T}}}]$, where ${\rm{\beta }}=\frac{{\eta }_{s}}{{\eta }_{s}+{\eta }_{p}}\,\,$is the ratio of the solvent viscosity *η*_*s*_ to the total of solvent viscosity and solute viscosity *η*_*p*_. *F*_0_ corresponds to the external body force and **i** represents the unit vector in the *x*-direction. ${\rm{Re}}=\frac{{U}_{max}H}{\rho {\eta }_{s}}$ is a dimensionless parameter, *H* is the characteristic length of the flow and equals to the half width of the channel. In the Newtonian fluid flow, there is no elastic component and **T**^*p*^ = 0. For the viscoelastic fluid described in the Oldroyd-B model, the elastic stress term in the constitutive equation is described as

3 $${{\bf{T}}}^{p}=\frac{1-\beta }{Re\cdot Wi}[{\bf{C}}-{\bf{I}}]$$

where **C** is the conformation tensor; **I** (Kronecker symbol) is the unit tensor; *η*_*P*_ is the dynamic viscosity of the solute, and $Wi=\frac{\lambda u}{L}$ is the Weissenberg number which describes the strength of the elasticity of the fluid, λ is the relaxation time of the viscoelastic fluid. The elastic stress term is a symmetric positive definite second-order tensor.

The conformation tensor transport equation is:

4 $$\frac{\partial {\bf{C}}}{\partial t}+({\bf{u}}\cdot \nabla ){\bf{C}}=(\nabla \cdot {\bf{u}}){\bf{C}}+{\bf{C}}{(\nabla \cdot {\bf{u}})}^{{\rm{T}}}+\frac{{\bf{C}}-{\bf{I}}}{Wi}$$

### Log-conformation reformulation

Numerical simulations for viscoelastic fluid flows at a high *Wi* number typically break down due to the computational instability. The instability may arise from the nonlinear term in Eq. (4) the hyperbolic characteristic of Eq. (4), an iterative algorithm, and the existence of equilibrium. The rapid growth rate of conformation tensor is inexact to polynomial fitting, resulting in divergence of calculation^37,38^. Instead of solving the elastic stress tensor or the conformation tensor, we solve the log-conformation tensor, where the extensional components of deformation field act additively rather than multiplicatively. After solving the log-conformation tensor, the conformation and elastic stress tensors can be recovered.

In this paper, we reconstructed the conformation tensor by the alternative exponential method, an algorithm that turns the nonlinear terms in the equations into linear terms. Specifically:

5 $$\frac{\partial {\bf{C}}}{\partial t}+({\bf{u}}\cdot \nabla ){\bf{C}}-(\nabla \cdot {\bf{u}}){\bf{C}}-{\bf{C}}{(\nabla \cdot {\bf{u}})}^{{\rm{T}}}=\frac{g({\bf{C}}){\rm{P}}({\bf{C}})}{Wi}$$

The velocity gradient can be expressed as $\nabla {\bf{u}}={\boldsymbol{\Omega }}+{\bf{B}}+{\bf{N}}{{\bf{C}}}^{-1}$, which decomposes velocity gradient into extensional component **B** and rotational components **Ω** and **N**. The **C**, **B**, **Ω**, **N**, and **D** are described as follows:

6 $$\begin{array}{ll}\begin{array}{rll}{\bf{C}} & = & {\bf{R}}(\begin{array}{lll}{k}_{1} & 0 & 0\\ 0 & {k}_{2} & 0\\ 0 & 0 & {k}_{3}\end{array}){{\bf{R}}}^{{\rm{T}}}\\ {\bf{N}} & = & {\bf{R}}(\begin{array}{lll}0 & {n}_{1} & 0\\ -{n}_{1} & 0 & {n}_{3}\\ -{n}_{2} & -{n}_{3} & 0\end{array}){{\bf{R}}}^{{\rm{T}}}\\ {\bf{R}}\nabla {\bf{u}}{{\bf{R}}}^{{\rm{T}}} & = & (\begin{array}{lll}{m}_{11} & {m}_{12} & {m}_{13}\\ {m}_{21} & {m}_{22} & {m}_{23}\\ {m}_{31} & {m}_{32} & {m}_{33}\end{array})\end{array} & \begin{array}{l}{\boldsymbol{\Omega }}={\bf{R}}(\begin{array}{lll}0 & {\omega }_{1} & 0\\ -{\omega }_{1} & 0 & {\omega }_{3}\\ -{\omega }_{2} & -{\omega }_{3} & 0\end{array}){{\bf{R}}}^{{\rm{T}}}\\ {\bf{B}}={\bf{R}}(\begin{array}{lll}{m}_{11} & 0 & 0\\ 0 & {m}_{22} & 0\\ 0 & 0 & {m}_{33}\end{array}){{\bf{R}}}^{{\rm{T}}}\\ {\bf{D}}=(\begin{array}{lll}{k}_{1} & 0 & 0\\ 0 & {k}_{2} & 0\\ 0 & 0 & {k}_{3}\end{array})\end{array}\end{array}$$

The conformation tensor transport equation can be transformed as follows:

7 $$\frac{\partial {\bf{C}}}{\partial t}+({\bf{u}}\cdot \nabla ){\bf{C}}-({\boldsymbol{\Omega }}{\bf{C}}-{\bf{C}}{\boldsymbol{\Omega }})-2{\bf{B}}{\bf{C}}=\frac{g({\bf{C}}){\rm{P}}({\bf{C}})}{Wi}$$

Here, we introduce a parameter **Ψ** associated with **R** and **Λ**, where **R** is the feature vector of **Ψ**, **Λ** is diagonal matrix constituted by the eigenvalues of **Ψ**.

8 $${\boldsymbol{\Psi }}={\bf{R}}{\boldsymbol{\Lambda }}{{\bf{R}}}^{{\rm{T}}}$$

9 $${\boldsymbol{\Psi }}=\,\mathrm{log}({\bf{C}})={\bf{R}}\,\mathrm{log}({\bf{D}}){{\bf{R}}}^{{\rm{T}}}$$

10 $${\bf{C}}={{\rm{e}}}^{{\boldsymbol{\Psi }}}={\bf{R}}\exp ({\boldsymbol{\Lambda }}){{\bf{R}}}^{{\rm{T}}}$$

Substituting Eqs (9) and (10) into Eq. (7), we get:

11 $$\frac{\partial {\boldsymbol{\Psi }}}{\partial t}+({\bf{u}}\cdot \nabla ){\boldsymbol{\Psi }}-({\boldsymbol{\Omega }}\cdot {\boldsymbol{\Psi }}-{\boldsymbol{\Psi }}\cdot {\boldsymbol{\Omega }})-2{\bf{B}}=\frac{g({{\rm{e}}}^{{\boldsymbol{\Psi }}}){{\rm{e}}}^{-{\boldsymbol{\Psi }}}P({{\rm{e}}}^{{\boldsymbol{\Psi }}})}{W{\rm{i}}}$$

The dominant nonlinear term of original conformation tensor in Eq. (4) turns into a linear term through strain rate tensor reconstruction in Eq. (11), which can significantly improve the computational stability. LCR-based solver performance has been verified in our previous study^39^.

### Numerical procedure

**Figure 2:**
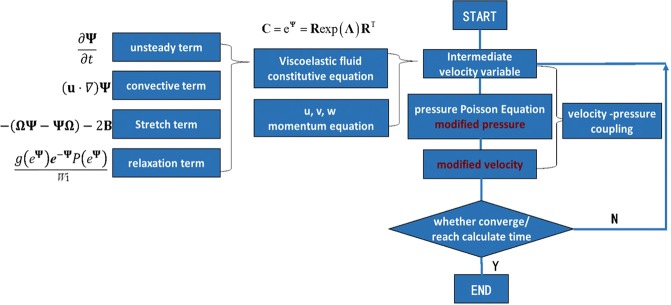
Process flow of the numerical simulation.

The numerical process is shown in Fig. 2. In the numerical calculation, an implicit finite-volume approach is used; the first-order Euler scheme is applied for time marching of all unsteady equations^40^ with the dimensionless time step δ*t* being 10^−3^. In spatial discretization, the QUICK scheme^41^ is adopted for the convective term in Eq. (2), and the MINMOD scheme^42^ are used for the logarithmic conformation tensor transport. The relationship between different external forces and the velocity response signal can be obtained from one-dimensional flow by comparative analysis.

## Results and Discussion

In this paper, we investigate the velocity response of viscoelastic fluid in the two-dimensional fully-developed Poiseuille flow under external forces in different forms, i.e., a constant force, a rectangular function force, and a square wave force.

### Velocity response of viscoelastic fluid flow under a constant force

In this section, we first compare the transient velocity responses between the Newtonian fluid and the viscoelastic fluid flows when a constant force is applied. We set the applied constant force *F*_0_ = 2 from time *t* = 0 and β is 0.01. The elasticity number, which is defined as the ratio of elastic stress to the inertial stress, or *E* = *Wi/Re*, is set as *E*_*N*_ = 0 for the Newtonian fluid and *E*_*V*_ = 1 for the viscoelastic fluid.

**Figure 3:**
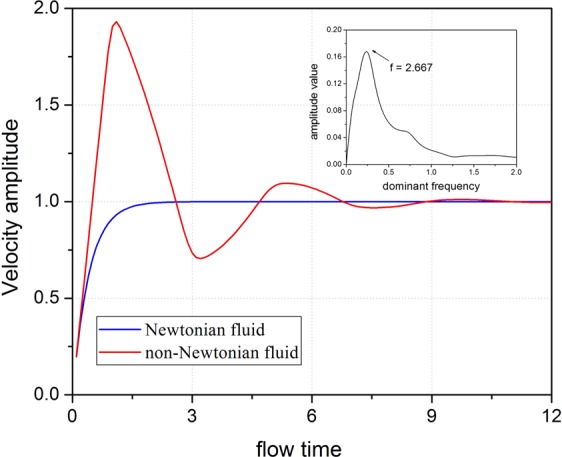
Velocity responses to constant force *F*_0_ = 2 on the Newtonian fluid flow with *E*_*N*_ = 0 and viscoelastic fluid flow with *E*_V_ = 1. *β* = 0.01. Insert figure: fast Fourier transform of the velocity of viscoelastic fluid flow.

The centerline velocities ***u*** (dimensionless) of two fluid flows at the outlet in a dimensionless time *t* are shown in Fig. 3. For the Newtonian fluid flow, the velocity *u*_*N*_ first increases logarithmically from time 0 to 1 and then saturates at *u*_*N*_ = 1 due to the balance between the applied force and the viscous dissipation in the flow. In contrast, the velocity of viscoelastic fluid *u*_*V*_ fluctuates as an under-damped vibration function with time: *u*_*V*_ first increases to a peak value of 1.9 at *t* = 1.5, drops to a dip value of 0.7 at *t* = 4.1, and then continues to oscillate with a damping effect until it finally stabilizes at *u*_*V*_ = 1. The oscillation originates from the non-zero elasticity of viscoelastic fluid responding to the constant *F*_0_. *u*_V_ increases at time 0 due to the applied *F*_0_. At the same time, the molecular microstructures in viscoelastic fluid are deformed due to its elasticity and elastic potential energy is stored in the molecular microstructures of viscoelastic fluid. The potential energy can be converted to kinetic energy which makes *u*_V_ continue to increase and exceed the stable velocity of the Newtonian fluid until time *t* = 1.5. And then, *u*_V_ starts to drop due to the viscous effect. Meanwhile, the kinetic energy of fluid flow converts to the potential energy, which makes *u*_V_ continue to drop below the stable velocity of the Newtonian fluid flow until *t* = 4.1. At this moment, *F*_0_ overwhelms the viscous dissipation and *u*_V_ starts to increase again. The process repeats which leads to an oscillation velocity response of viscoelastic fluid flow to the constant *F*_0_. During the oscillation, the total energy decreases due to the viscosity, and therefore the oscillation is damping. The damped oscillation of velocity response in viscoelastic fluid flow reaches a stable state when the viscous resistance balances with the external *F*_0_ and the molecular microstructure in viscoelastic fluid remains in a static state. Then, viscoelastic fluid flows at a constant velocity *u*_V_ = 1 after *t* = 15. The stable velocities are the same for the Newtonian fluid and viscoelastic fluid flows with the same viscosity under the same constant force. Further investigation on the damped oscillation in the frequency domain reveals a peak resonance at *f* = 0.27 based on fast Fourier transform, as shown in the inset of Fig. 3.

**Figure 4:**
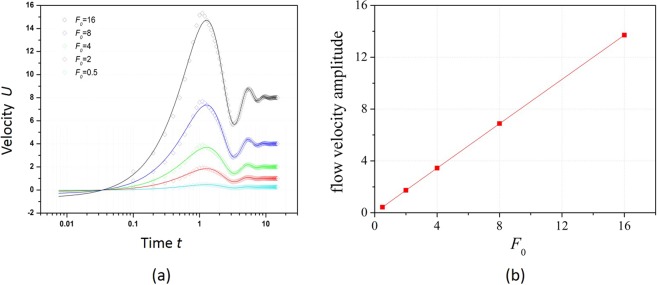
(**a**) Centerline velocity of viscoelastic fluid flow and (**b**) the amplitude of velocity under different constant forces. The circles in (**a**) represent the numerically simulated results, and the solid line is the fitted value *A* in Eq. (12).

When the constant forces *F*_0_ with different magnitudes are applied to viscoelastic fluid flows, the velocity oscillates with an amplitude proportional to *F*_0_ while the oscillation peaks and dips occur at the same time value for all *F*_0_, as indicated by open circles in Fig. 4(a). Here, the oscillation is assumed to be a damped harmonic wave function and is expressed as

12 $$u(t)={u}_{0}+A{e}^{-\alpha t}\,\sin (\frac{2\pi }{T}(t-{t}_{c}))$$

By fitting parameters in Eq. (12) to the numerically calculated values for different *F*_0_, the oscillation curves of *u* are shown as solid lines in Fig. 4(a). The fitted curves closely match with numerical results, confirming that the velocity oscillates in a damped manner. The parameters are derived for different *F*_0_ as shown in Table 1, where *A* and *T* are the amplitude and period of harmonic oscillation, respectively; *α* is the damping coefficient; *u*_0_ and *t*_c_ are the offset values for the amplitude and time, respectively. *α* and *T* remain the same when *F*_0_ changes because they are intrinsic properties of viscoelastic fluid and only affected by the viscosity and elasticity of viscoelastic fluid. The amplitude of sinusoid functions *A*, on the other hand, increases proportionally with *F*_0_, which is plotted in Fig. 4(b). The time offset *t*_c_, which relates to the oscillation phase of velocity, is the same for different *F*_0_ because the starting time of force is the same.

**Table 1:** The fitting coefficients for transient velocity response of viscoelastic fluid under different constant forces.

	t_c_	T	α	A
*F* = 0.5	0.45	4.18	0.51	0.43
*F* = 2	0.45	4.18	0.51	1.73
*F* = 4	0.45	4.18	0.51	3.45
*F* = 8	0.45	4.16	0.52	6.88
*F* = 16	0.45	4.04	0.52	13.7

**Figure 5:**
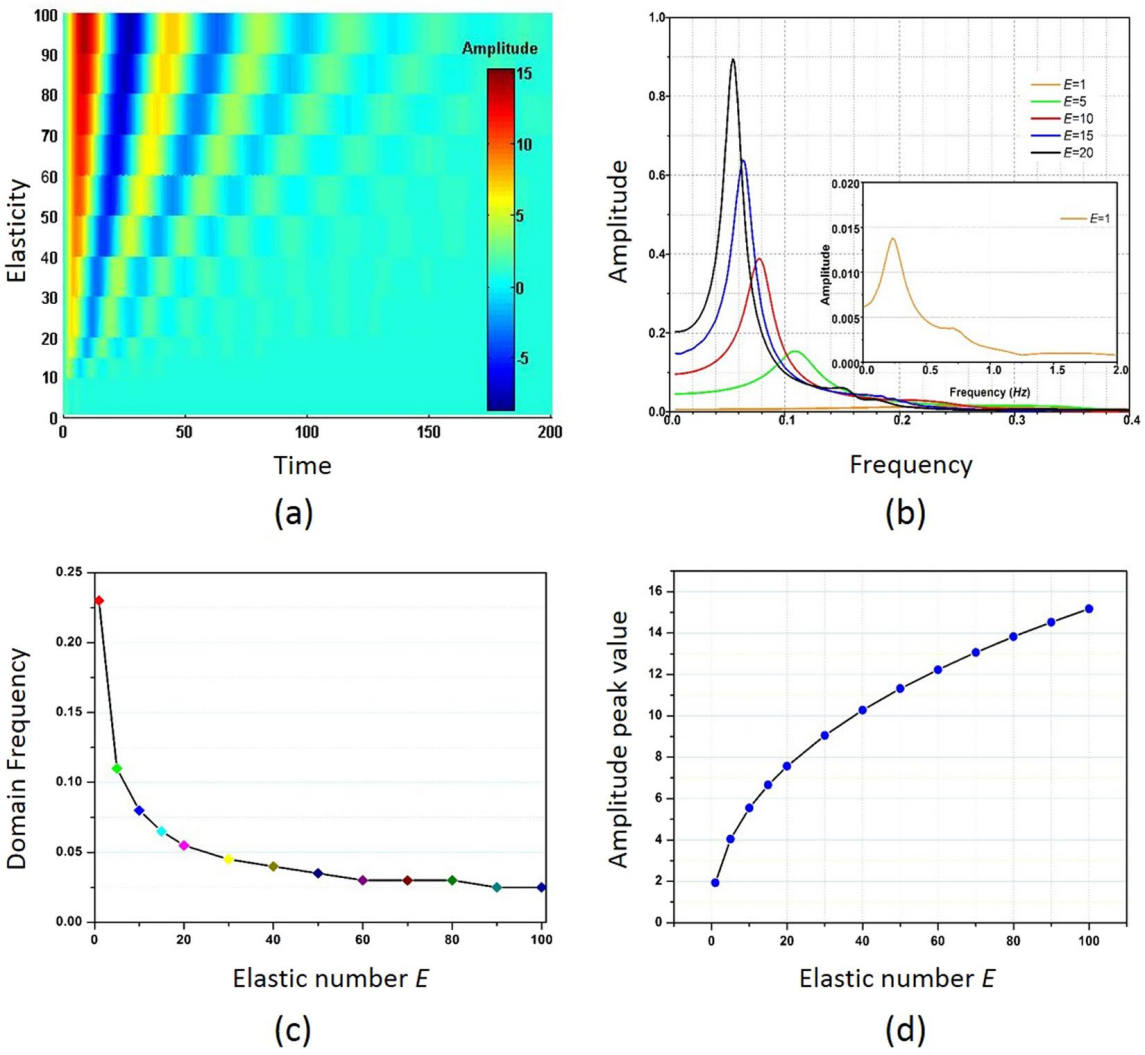
(**a**) Elasticity number dependent velocity of viscoelastic fluid flow with viscosity *β* = 0.01 response to a constant force *F*_0_ = 2; (**b**) frequency domain of the temporal response; (**c**) amplitude of the first velocity peak; (**d**) drop of domain frequency as elasticity number increases.

As analyzed above, the damped oscillation depends strongly on the elasticity of viscoelastic fluid. In a fluid with larger elasticity, more potential energy can be stored in the fluid before it is saturated. The potential energy is then converted to the kinetic energy and drives the velocity to increase for much time. For the same reason, with larger elasticity, more time is also needed for the fluid to convert the kinetic energy to the potential energy during the velocity drop period. As a result, the oscillation period should be longer for the fluid with a larger elasticity number. This is numerically confirmed by comparing the transient response of centerline velocities at different elasticity numbers as shown in Fig. 5(a). The colour bar represents the velocity amplitude. It is found that the fluid with larger elasticity oscillates slower than the fluid with less elasticity. Also, the oscillation amplitude is larger for the fluid with larger elasticity because more energy can be stored in this fluid and be converted to the kinetic energy.

The velocity response at different elasticity numbers is then fitted using Eq. (12) and the coefficients are shown in Table 2. The oscillation period *T* increases from 4.16 to 17.94 as elasticity number *E* increases from 1 to 20. This proportional relation is explained quantitatively previously. The amplitude *A* also increases significantly from 1.79 to 7.26, which is because of a viscoelastic fluid with larger elasticity gains and converts more potential energy to the kinetic energy. In addition, the effective damping factor *T· α*, or the damping in one oscillation period, decreases from 2.20 to 0.66, indicating that the fluid with larger elasticity will experience a relatively lower damping effect under a constant force excitation.

**Table 2:** The fitting coefficients of for transient velocity response of viscoelastic fluid under different *E*.

	t_c_	T	α	T· α	A
*E* = 1	0.46	4.16	0.529	2.20	1.79
*E* = 5	0.46	9.00	0.122	1.10	3.70
*E* = 10	0.47	13.70	0.063	0.80	5.19
*E* = 15	0.47	14.53	0.046	0.67	6.32
*E* = 20	0.47	17.94	0.037	0.66	7.26

The oscillation is further investigated in the frequency domain through a fast Fourier transform of temporal response. Different resonant frequencies of *f* = 0.242, 0.110, 0.078, 0.064 and 0.055 are obtained for *E* = 1, 5, 10, 15 and 20, respectively, as shown in Fig. 5(b). We refer to this resonant frequency as the dominant frequency of viscoelastic fluid. The dominant frequency drops as the elasticity number increases, as seen in Fig. 5(c), which results from the increase of oscillation period. Meanwhile, the peak amplitude of velocity increases with the elasticity number, as shown in Fig. 5(d). This trend is caused by the more kinetic energy converted to the potential energy during the molecular deformation process and more potential energy converted to the kinetic energy during the molecular release process.

**Figure 6:**
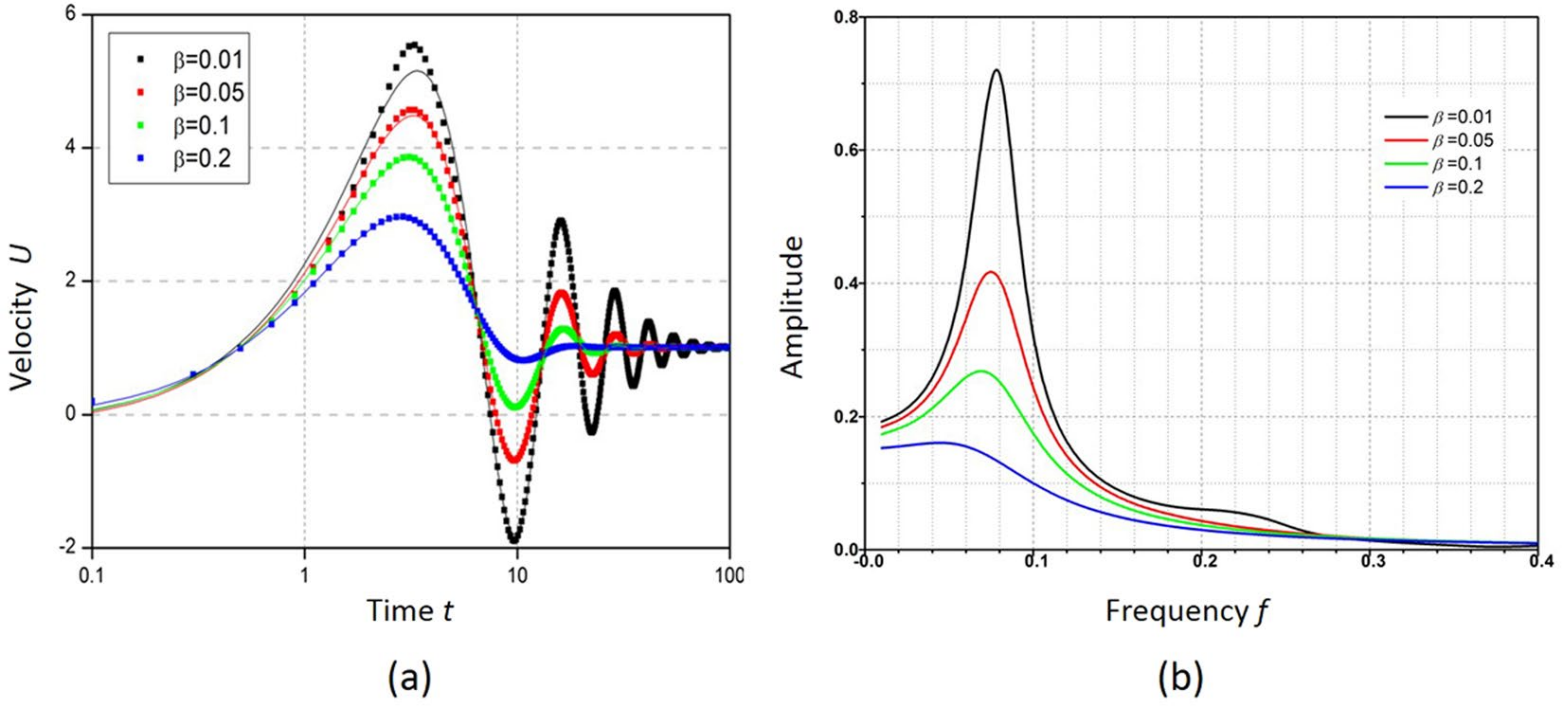
(**a**) Centerline velocity for viscoelastic fluid flow with *E* = 10, *F*_0_ = 2 and different *β*; (**b**) the corresponding Fourier transform of transient velocity.

Besides elasticity, the viscosity is another critical property of viscoelastic fluids which describes its resistance to gradual deformation under shear stress or tensile stress. This resistance induces friction, which slows down the flow velocity. The centerline velocities are compared for viscoelastic fluids with a fixed elasticity number *E* = 1 and different *β* in Fig. 6(a). As expected, the oscillation amplitude of velocity drops faster in viscoelastic fluid with larger viscosity than that in one with lower viscosity. It can be also observed that the peaks and dips of oscillation occur at the same time interval regardless of viscosity. Therefore, the oscillation period does not change with the viscosity, but the damping behaviour does. The fitting coefficients obtained using Eq. (12) for different *β* are listed in Table 3. The oscillation period *T* and the amplitude *A* remain almost the same when *β* increases from 0.01 to 0.2. In contrast, the damping factor *α* increases significantly from 0.06 to 0.29 for the same values of *β*, suggesting that the oscillation damping depends remarkably on the viscosity. The dominant frequency is the same for the viscoelastic fluid with different *β* as shown in Fig. 6(b) because their oscillation period is not affected, however, the amplitude at the dominant frequency is the highest for the fluid with the lowest *β*, which is due to the lowest energy loss in the oscillation.

**Table 3:** The fitting coefficients for transient velocity response of viscoelastic fluid under different *β*.

	t_c_	T	α	A
*β* = 0.01	0.47	13.68	0.06	5.18
*β* = 0.05	0.49	13.88	0.11	5.15
*β* = 0.1	0.49	13.38	0.17	5.14
*β* = 0.2	0.50	15.62	0.29	5.56

### Velocity response of viscoelastic fluid flow under a rectangular function force

**Figure 7:**
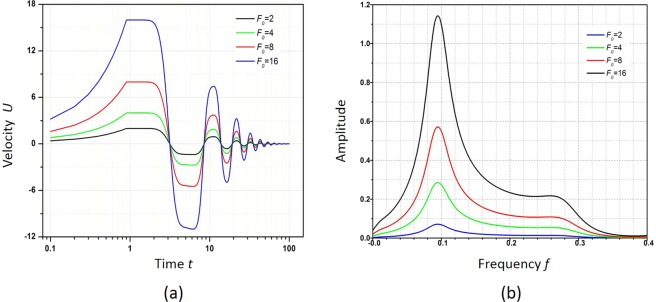
(**a**) Velocity response of viscoelastic fluid flow to the rectangular force with different amplitudes applied with the same unloaded time *T*_r_ = 1; (**b**) the Fourier transform of the response in (**a**).

In this section, we study the transient velocity response of viscoelastic fluid flow with elasticity *E* = 1 to an applied rectangular function force. Here, we define the force as:

13 $${F}_{0}=\{\begin{array}{ll}0 & (t < 0)\\ {F}_{r} & (0\,\leqq \,t\,\leqq \,{T}_{r})\\ 0 & (t > {T}_{r})\end{array}$$

where *T*_r_ is the force applied time. Figure 7(a) shows the transient velocity response to the rectangular force with different *F*_0_ and fixed *T*_r_ = 1. The velocity increases from time *t* = 0 to *t* = *T*_r_, and during the period, the condition is the same with one when a constant force is applied. As discussed previously, under a constant force, the velocity oscillation for the fluid with *E* = 1 has a period *T* = 13.7 and is in its ramp-up stage of the first cycle when t = *T*_r_. However, for the rectangular force, the force is unloaded at this point. One interesting phenomenon observed is that the velocity remains constant for another 1 s after the force is unloaded until *t* = 2. After that, the velocity amplitude starts to drop sharply from *t* = 2 to *t* = 4 and then drop very slowly from *t* = 4 to *t* = 6. Then it becomes damped oscillation and finally ends with a velocity of 0. The length of this “platform effect”, the short-term constant velocity behaviour after the force is unloaded, is independent of the rectangular force amplitude but the magnitude of velocity increases proportionally with the amplitude. A fast Fourier transform is performed to further investigate the velocity response, as shown in Fig. 7(b). Responses to rectangular forces of all amplitudes exhibit a resonance frequency *f* = 0.091 Hz, which is blue shifted compared to the resonance frequency *f* = 0.078 Hz under the constant force excitation condition as indicated in Fig. 6.

**Figure 8:**
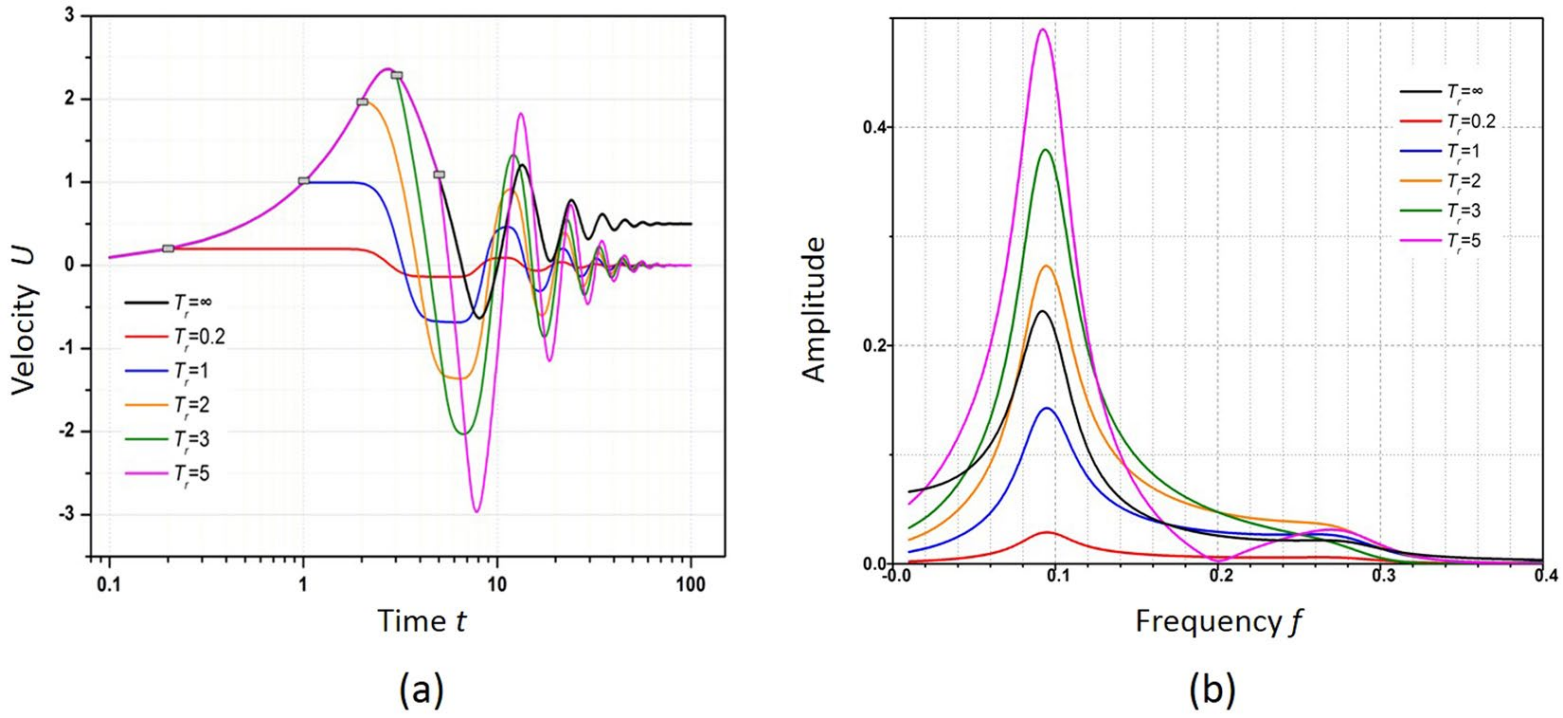
(**a**) Velocity response of viscoelastic fluid flow to the rectangular force with the same amplitude *F*_0_ = 2 applied with different unloaded time *T*_r_; (**b**) the Fourier transform of the response in (**a**).

By using viscoelastic fluid with a larger elasticity (*E* = 10) and varying *T*_r_, it continues to study the platform effect. It is found that the platform effect only appears when the force is unloaded during the velocity ramping stage of the first oscillation period, as seen in Fig. 8(a). At this stage, the molecular microstructure of viscoelastic fluid is stretched under large shear stress if an external force is applied and potential energy is stored in viscoelastic fluid. Due to the memory characteristic of viscoelastic fluid, the potential energy releases, and the velocity is maintained at the same value until the conversion from the potential energy can no longer support the flow at this velocity, which will, therefore, start to drop afterwards. The energy conversion time is related to the elasticity of viscoelastic fluid, which ends at the ¼ period, or the peak value time of the first oscillation. Therefore, it can be concluded that a viscoelastic fluid with larger elasticity will display a longer platform effect due to the longer period. In the fast Fourier transform of the response, resonance occurs at the same frequency value of *f* = 0.091 Hz for different *T*_r_, which indicates that the domain frequency does not change with *T*_r_. In contrast, the power spectra density at the resonant frequency changes significantly when the unloading time changes. Therefore, the rectangular force does not affect the domain frequency but only determines the resonant amplitude.

### Velocity response of viscoelastic fluid flow under a square wave force

**Figure 9:**
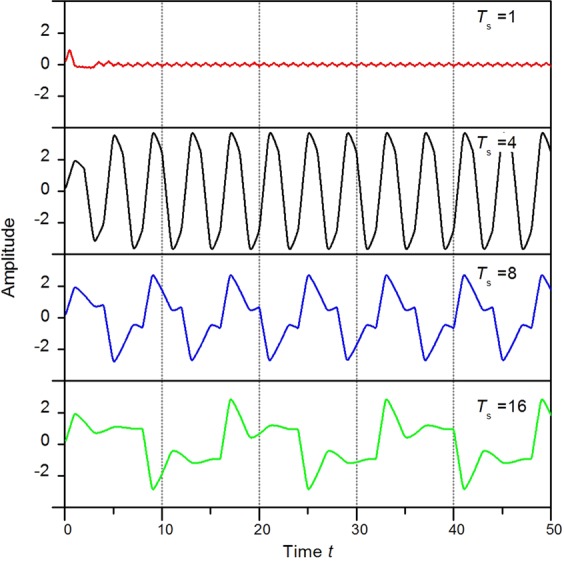
Velocity responses of viscoelastic fluid flow to the applied square wave force with periods of 1, 4, 8 and 16 s, respectively.

In this section, a square wave force *F*_s_ is applied to the viscoelastic fluid, which is defined as

14 $${F}_{0}(t)=\{\begin{array}{ll}1 & (0\le t < \frac{{T}_{s}}{2})\\ -1 & (\frac{{T}_{s}}{2}\le t < {T}_{s})\\ {F}_{0} & (t+{T}_{s})\end{array}$$

where *T*_*s*_ is the period of the square wave. We investigate the velocity response to the square wave force on a viscoelastic fluid with elasticity *E* = 1. Here, square wave forces with period *T*_*s*_ = 1, 4, 8 and 16 are applied to the viscoelastic fluid, and the velocity response is shown in Fig. 9. For *T*_*s*_ = 1, 8 and 16, multiple resonance peaks are observed. These peaks deviate greatly from the intrinsic period of the velocity oscillation *T*_*i*_ = 4.16, which is found earlier for a viscoelastic fluid under a constant force (as shown in Table 2). At *T*_*s*_ = 4, which is close to *T*_*i*_, each period has a single peak, which is consistent with single resonance.

**Figure 10:**
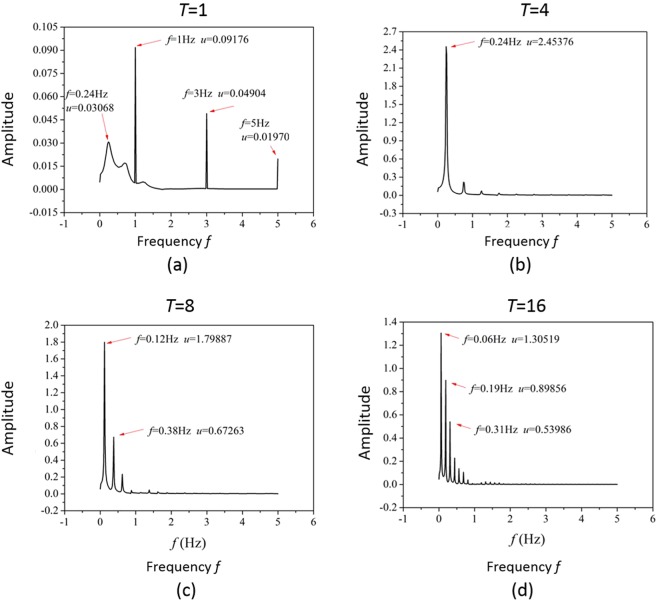
Frequency responses of viscoelastic fluid flow to the applied square wave force with periods of 1, 4, 8 and 16 s, respectively.

We further study the response of viscoelastic fluid flow to a square wave force in the frequency domain. A Fourier transform is applied to each velocity response for square waves with periods of 1, 4, 8 and 16 s. The applied square wave excitation is an odd function and its corresponding Fourier expansion can be written as^39^

15 $${F}_{s}(t)=\frac{4}{\pi }\sum _{n=1,3,\mathrm{5..}}^{\infty }\frac{1}{n}\,\sin (2\pi nft)$$

where *f* = 1/*T*_s_. Therefore, resonant peaks are induced at *n*·*f*, where *n* is equal to 1, 3, 5 and so on. For example, resonant peaks appear at 1, 3, and 5 Hz when *T*_*s*_ = 1 with a corresponding *f* of 1 Hz as shown in Fig. 10(a). Similarly, for *T*_s_ = 8 and the corresponding *f* = 0.125 Hz, resonant peaks appear at *f* (0.12 Hz), 3 *f* (0.38 Hz) and 5 *f* (0.63 Hz), as shown in Fig. 10(c). By the same mechanism, at *T*_*s*_ = 16 s or *f ≈*0.06 Hz, resonant peaks are induced at *f* (0.06 Hz), 3 *f* (0.19 Hz) and 5 *f* (0.31 Hz) and so on. However, when *T*_*s*_ is close to the natural period of viscoelastic fluid, or 4 s, other order frequencies are significantly suppressed, and only the first resonant mode at *f* = 0.24 Hz is strongly induced, as shown in Fig. 10(b). This is because the stimulus frequency matches the natural frequency and most of the energy is coupled at this frequency. As a result, the resonant amplitude is also much larger than that in other cases. Because the domain frequency of viscoelastic fluid flow can be tuned just by changing the corresponding elasticity, such fluids can be readily used for the narrowband filtering and amplification of signals. This is especially useful in biochemical applications due to fluid compatibility.

## Conclusions

We investigate the transient velocity response of viscoelastic fluid flow to externally applied forces based on numerical simulations. The velocity responses to different forces are interpreted which highly depend on the intrinsic properties of viscoelastic fluid, particularly its elasticity and viscosity. Some interesting results are obtained as follows:

1. A damped oscillation velocity response to a constant force is obtained. The oscillation amplitude, period and damping coefficient can be modulated by changing the viscosity coefficient *β* and the elasticity *E* of the viscoelastic fluid. It demonstrates that the period of the oscillation signal relies on the elasticity number of the fluid while the damping coefficient is dependent on viscosity.
2. We investigate the velocity response to a rectangular force and a platform effect in a viscoelastic fluid was observed, by which the velocity is maintained at a constant value for certain time after the force is unloaded.
3. A periodic square wave force is also applied and harmonic resonance is occurred when the applied force period matches the intrinsic period of the viscoelastic fluid. The resonance is due to the coupling between the force and the fluid and the resonant amplitude of the velocity is maximized.

By analyzing the response features of viscoelastic fluid excited by external forces, the transient velocity can be utilized as modulated signal when processing different force functions in microfluidic circuit. In general, the nonlinear response to the external force allows the viscoelastic fluid to be widely applied in the waveform modulation and filter applications, such as digital signal processing, signal converter, controller, signal encryption, password converter, etc.
